# Psychometric Properties of the Diabetes Management Self-Efficacy Scale in Korean Patients with Type 2 Diabetes

**DOI:** 10.1155/2015/780701

**Published:** 2015-05-18

**Authors:** Eun-Hyun Lee, Jaap van der Bijl, Lillie M. Shortridge-Baggett, Seung Jin Han, Seung Hei Moon

**Affiliations:** ^1^Graduate School of Public Health, Ajou University, 164 Worldcup-ro, Yeongtong-gu, Suwon-si, Gyeonggi-do 443-380, Republic of Korea; ^2^Inholland University of Applied Sciences, De Boelelaan 1109, 1081 HV Amsterdam, The Netherlands; ^3^Department of Graduate Studies, Lienhard School of Nursing, Pace University, 861 Bedford Road, Pleasantville, NY 10570-2799, USA; ^4^Department of Endocrinology and Metabolism, School of Medicine, Ajou University, Suwon, Gyeonggi-do 443-380, Republic of Korea; ^5^Department of Nursing, Graduate School, Inha University, 100 Inha-ro, Nam-gu, Incheon 402-751, Republic of Korea

## Abstract

*Objectives*. The aims of this study were to perform a cultural translation of the DMSES and evaluate the psychometric properties of the translated scale in a Korean population with type 2 diabetics. *Methods*. This study was conducted in patients with diabetes recruited from university hospitals. The first stage of this study involved translating the DMSES into Korean using a forward- and backward-translation technique. The content validity was assessed by an expert group. In the second stage, the psychometric properties of the Korean version of the DMSES (K-DMSES) were evaluated. *Results*. The content validity of the K-DMSES was satisfactory. Sixteen-items clustered into four-subscales were extracted by exploratory factor analysis, and supported by confirmatory factor analysis. The construct validity of the K-DMSES with the Summary of Diabetes Self-Care Activities scale was satisfactory (*r* = 0.50, *P*<0.001). The Cronbach's alpha and intraclass correlation coefficient were 0.92 and 0.85 (*P*<0.001; 95% CI = 0.75–0.91), respectively, which indicate excellent internal consistency reliability and test-retest reliability. *Conclusions*. The K-DMSES is a brief instrument that has demonstrated good psychometric properties. It is therefore feasible to use in practice, and is ready for use in clinical research involving Korean patients with type 2 diabetes.

## 1. Introduction

The prevalence of diabetes has reached an almost epidemic level. About 382 million people in the world have diabetes, and this number is expected to rise to 592 million by 2035 [1]. The prevalence of diagnosed diabetes in Korea has increased from 2% in the 1970s to 9.8% in 2011, and in 2012 the rate for patients with poor glycemic control was reportedly as high as 71.5% [2]. These findings suggest the presence of a substantial financial burden on the Korean health-care system.

Diabetes can be substantially improved by performing tasks such as taking prescribed medications, monitoring blood glucose levels, eating an appropriate diet, and exercising regularly. These are all day-to-day behaviors that patients must carry out to control their disease, a process that is termed self-management [3]. The traditional approach to diabetes self-management has been to educate patients about the disease and provide them with the skills necessary to control it [4]. According to one systematic review, although such self-management education appears to be successful, it exerts only small-to-moderate effects on the diabetes [5]. Diabetes researchers insist that providing patients with knowledge and skills is crucial, but these approaches appear to be insufficient for including the required behavioral changes among patients with diabetes [6, 7]. Therefore, further factors that contribute to more effective diabetes self-management need to be considered.

Self-efficacy, a term that is derived from the social cognitive theory, refers to “belief in one's capability to organize and execute the course of action required to produce given levels of attainments” [8]. Self-efficacy influences the individual's choice of behaviors; people tend to engage in tasks when they feel competent to perform them and to avoid them when they feel that they exceed their capabilities. Self-efficacy also influences how people motivate themselves in the tasks that they undertake. That is, people with a strong sense of self-efficacy view their tasks or behaviors as challenges to be mastered, even if they are difficult. Efficacious people tend to set challenging goals and maintain commitment to them. In addition, self-efficacy beliefs influence emotional states; people with higher self-efficacy are likely to have reduced stress levels and lower risks of depression than those with low self-efficacy [9]. Thus, self-efficacy has emerged as a crucial factor in diabetes self-management behaviors [10–12].

Instruments that measure self-efficacy are broadly categorized into general and specific types of scales. Some researchers view self-efficacy as a more trait-like general construct, referring to one's overall competence to perform across a variety of different situations [13, 14]. Instruments developed based on this perspective are general self-efficacy scales. Others state that self-efficacy judgments are specific to behaviors and the situations in which those behaviors occur [15, 16]; that is, people perceive different levels of capability of performing in particular domains or situations of functioning. Instruments developed from this conceptualization are specific self-efficacy scales. Patients with diabetes must perform particular tasks to control their blood glucose in order to prevent complications. They may possess a high self-efficacy with respect to taking medication, but a low self-efficacy regarding physical exercise. Scales that are specifically designed for patients with diabetes are therefore more appropriate for measuring their self-efficacy [10, 17, 18].

There have been previous attempts in Korea to develop a specific scale measuring the perceived self-efficacy of diabetes self-management [19, 20], but they have produced only a primitive stage of scale development; the items were derived from the literature without verifying their psychometric properties. Applying such instruments in the studies for clinical interventions may threaten the reliability of their outcomes. The Diabetes Management Self-Efficacy Scale (DMSES) is a specific-type instrument that was developed by the members of the International Partnership in Self-Management and Empowerment [21]. Its psychometric properties were found to be acceptable for populations with type 2 diabetes in several countries: Netherlands [21], United Kingdom [22], Australia [23], Turkey [24], and Taiwan [25]. However, these psychometric studies had methodological and statistical problems related to factors such as sample size, item redundancy, and the underlying constructs. With these issues in mind, the aims of the present study were to perform a culture-sensitive translation of the DMSES and then evaluate the psychometric properties of the translated scale in a Korean population with type 2 diabetes.

## 2. Methods

### 2.1. Step  I: Cultural Translation and Content Validity

The English-language version of the DMSES was translated into Korean using a forward and backward translation technique, based on the guidelines of Brislin [26]. A bilingual health professional and a layperson independently translated the English version into Korean using semantic equivalence. An expert panel of three bilinguals checked the two potential Korean versions and achieved a consensus on a Korean version. The Korean version was then independently translated back into English by another two bilinguals. The panel checked the back-translated versions against the original English version. Any discrepancies between the translated and original English versions were either confirmed by one of the original developers or else a consensus was reached by the panel. The preliminary Korean version was thus produced, and the Korean version was finalized after one professor majored in Korean literature had reviewed its grammar.

Five experts (one physician, one professor in nursing, and three diabetes educators) were involved in assessing the content validity of the final Korean version of the DMSES (K-DMSES). These experts were asked to rate each item of the preliminary K-DMSES whether they considered it “essential,” “useful, but not essential,” or “not essential” [27]. In addition, they were asked to answer open questions regarding whether or not there were any ambiguous words, jargon, or value-laden words and whether or not there were items that needed to be modified.

### 2.2. Step  II: Psychometric Evaluation of the K-DMSES

#### 2.2.1. Participants and Procedures

This was a methodological study to assess the psychometric properties of the K-DMSES. A convenience sample of 440 patients with type 2 diabetes was recruited from two university hospitals in South Korea. This sample size satisfied the requirement that at least 7 times the total number of items is needed for psychometric tests [28]. The inclusion criteria for the participants were being aged at least 20 years, being diagnosed with diabetes type 2, and being articulate in the Korean language. The participants were asked to sign a consent form and complete a package of questionnaires. Of these, 70 were given an envelope containing the K-DMSES questionnaire for the assessment of test-retest reliability. They were asked to take it home and complete it 10 days later; a time interval of 1-2 weeks between repeated measures is often recommended [28]. Each participant was asked to post the return envelope containing the completed questionnaire near home.

#### 2.2.2. Ethical Consideration

Prior to data collection, this study was approved by the institutional review boards at the participating institutions. Participants were voluntary and those who agreed to participate signed a consent form. All participants were assured of their confidentiality.

#### 2.2.3. Questionnaires

The DMSES [21] is a self-reported questionnaire that comprises 20 items with 4 subscales: nutrition specific and weight, nutrition general and medical treatment, physical exercise, and blood sugar. Originally, each item was scored on a 5-point scale, but this was later revised to an 11-point scale on the UK English-language version [22]. Possible scores range from 0 to 200, with higher scores reflecting higher self-efficacy. The DMSES satisfied the content validity, factorial construct validity, internal consistency reliability, and test-retest reliability when it was developed. The English-language version of the DMSES, which was obtained from the developer, was translated into Korean and used in this study.

Based on previous studies [12, 25], it was hypothesized in this study that the DMSES was positively and moderately correlated with the Summary of Diabetes Self-Care Activities Scale (SDSCA) [29]. Therefore, the Korean version of the SDSCA was administered to test hypothesis testing construct validity. The SDSCA assesses the frequency of behavioral tasks in five aspects of the diabetes regimen: diet, exercise, self-monitoring of blood glucose, foot care, and smoking for the previous 7 days. The reliability and validity of the SDSCA, which comprises 11 items, were culturally adapted for Korean patients with type 2 diabetes [30, 31].

#### 2.2.4. Statistical Analyses

Statistical analyses were completed using the PASW (version 18) statistical package. General characteristics and missing data were calculated using descriptive statistics. The zero-order correlation matrix among the K-DMSES items was computed using Pearson's analysis.

A cross-validation approach involving both exploratory factor analysis (EFA) and confirmatory factor analysis (CFA) was used for the factorial construct analysis, and for the cross-validation, 440 patients were split into 2 subsamples using a random-sampling function of the computer program (Table 1). The homogeneity of the subsamples with regard to general characteristics was computed using *χ* ^2^ or Fisher's exact test. With subsample 1, Bartlett's test of sphericity and the Kaiser-Meyer-Olkin (KMO) measure of sampling adequacy were screened to justify undertaking EFA [32]. Then, EFA was performed using principal-axis factor analysis with Varimax rotation. Factors with an eigenvalue higher than 1 were retained, and the factor loading criterion was set at ≥0.4 [33]. For the CFA with subsample 2, a maximum-likelihood estimation procedure was performed. Multiple criteria were used to evaluate the model fit: the ratio of the *χ* ^2^ value to the degrees of freedom (CMIN/DF), goodness-of-fit index (GFI), standardized root-mean-square residual (SRMR), root-mean-square error of approximation (RMSEA), comparative fit index (CFI), and normed fit index (NFI). The following criteria were used to confirm that a model was an acceptable fit: relative CMIN/DF < 3, GFI > 0.9, SRMR < 0.08, RMSEA < 0.08, CFI > 0.9, and NFI > 0.9 [33–36].

Construct validity by means of the hypothesis testing approach was examined for the entire sample using Pearson's correlation analysis. Internal consistency reliability and test-retest reliability were evaluated using Cronbach's alpha and the intraclass correlation coefficient (ICC), respectively.

## 3. Results

### 3.1. Step  1: Cultural Translation and Content Validity

In Korean culture, workers often go out after work to socialize, either formally or informally, as a release from their job-related stresses, and this socializing often involves eating grilled meats or rice and drinking alcohol. It is difficult for a worker at a group dinner to refuse to eat or drink or to order other foods for only himself/herself. Thus, the term “company dinner” was added in the translation process to item 16: “… able to follow a healthy eating pattern when I am eating out, at a party, or at a company dinner.” Clinicians in Korea usually recommend that patients with diabetes visit their physicians every 3 months, based on the guidelines of the Korean Diabetes Association [37]. Therefore, item 18 (“… able to visit my doctor once a year to monitor my diabetes”) was changed to “… able to visit a clinic or a public health center four times a year to monitor my diabetes.”

With respect to the content validity, all of the experts considered all of the items to be essential. However, item 11 (“… able to exercise more if the doctor advises me to”) was refined by replacing the term “doctor” in this item with “health professional,” since patients with diabetes in Korea receive advice not only from physicians but also from diabetes educators (e.g., nurses or nutritionists). Three experts commented that there were content similarities between items 4 and 5 and between items 13 and 14; however, no deletions were performed at this stage. The experts recommended additional quantitative analysis. All 20 items were retained for the next step of psychometric evaluation.

### 3.2. Step  2: Psychometric Evaluation of the K-DMSES

#### 3.2.1. Missing Data

The rate of missing values was 0.23% for each of items 3, 11, and 15; these missing values were replaced by the mean value for each item. There were no missing values for any of the other items.

#### 3.2.2. Zero-Order Correlation Matrix

In the 20 × 20 zero-order correlation matrix, items 4/5, 14/13, and 16/15 were strongly correlated (*r* = 0.80–0.90), as expected from the results for content validity. These strong correlations indicate the presence of redundancy [38], and hence only one item of each pair was retained. Items 4 and 14 were retained because their contents are more specific to diabetes than those of items 5 and 13. Furthermore, item 16 (“eat out, at a party, or at a company dinner”) occurs more frequently in daily life than the content of item 15 (“eat on holiday”), and so item 16 was retained. Thus, items 5, 13, and 15 were deleted in order to remove content redundancy.

#### 3.2.3. Factorial Construct Validity

The general characteristics did not differ between subsamples, as assessed by *χ* ^2^ or Fisher's exact test (Table 1). With the randomly split subsample 1, the KMO statistic (0.89) and Bartlett's sphericity (*χ* ^2^ = 2602.62, *P* < 0.001) indicated that the correlation matrix was suitable for factor analysis. The initial EFA extracted a four-factor solution (eigenvalue > 1, Table 2), which accounted for 65.81% of the total variance. Item 7 was not significantly loaded on any factors at a criterion of > 0.40. EFA was conducted after deleting that item (Table 2), again yielding a four-factor solution that explained 67.28% of the total variance in all items. All items were significantly loaded onto one of four factors. There was no significant cross-loading of items on the factors. Factors 1–4 were labeled “nutrition” (items 4, 9, 10, 14, 16, and 17), “physical exercise/body weight” (items 6, 8, 11, and 12), “medical treatment” (items 18, 19, and 20), and “blood sugar” (items 1, 2, and 3).

**Figure 1:**
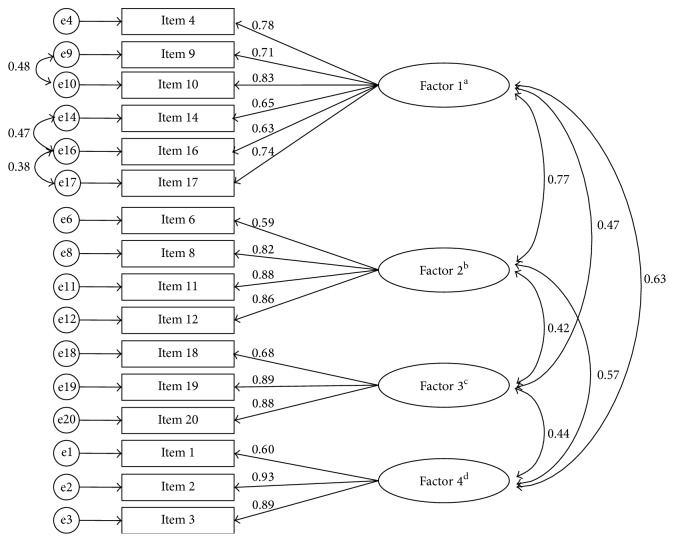
Confirmatory factor analysis for the Korean version of the Diabetes Management Self-Efficacy Scale. e: error term. ^a^Factor 1: nutrition. ^b^Factor 2: physical exercise/body weight. ^c^Factor 3: medical treatment. ^d^Factor 4: blood sugar.

To cross-validate the 16-item, 4-factor construct, CFA was conducted with the randomly split subsample 2. The SRMR value indicated an acceptable model fit, where the values of the other indexes indicated a poor-fitting model (Model 1, Table 3). Thus, the possibility of model modification was explored using modification indices (MIs) [39], which revealed that the MI value of pairing of error terms between items 14 and 16 was the largest, at 57.38. After modifying the covariance between the error terms of items 14 and 16 (Model 2), the model fit was significantly improved (Δ*χ* ^2^(1) = 66.51, *P* < 0.05). However, the values of some model-fit indexes (CMIN/DF, GFI, and RMSEA) were unsatisfactory, and there was still a large MI value (36.63) between the error terms of items 16 and 17. With this modification, CFA produced a significantly improved Model 3 (Δ*χ* ^2^(1) = 40.56, *P* < 0.05). After the final modification of the covariance between the error terms of items 9 and 10 (MI = 17.06), Model 4 was significantly improved compared with Model 3 (Δ*χ* ^2^(1) = 31.39, *P* < 0.05), and the values of all goodness-of-fit indexes, except GFI, were satisfactory. All items loaded meaningfully onto factors with standardized values ranged from 0.59 to 0.93 (Figure 1).

#### 3.2.4. Hypothesis Testing Construct Validity with the Total Sample

The K-DMSES score was moderately correlated with the SDSCA score (*r* = 0.50, *P* < 0.001), as hypothesized for the construct validity.

#### 3.2.5. Internal Consistency Reliability with the Total Sample

Overall Cronbach's alpha of the K-DMSES was 0.92, which indicates excellent internal consistency reliability. Cronbach's alpha values for the subscales of nutrition, physical exercise/body weight, medical treatment, and blood sugar were 0.89, 0.87, 0.86, and 0.84, respectively, which were all above the acceptability criterion of ≥0.70 [40].

#### 3.2.6. Test-Retest Reliability

Of the 70 patients who were asked to complete the K-DMSES twice, 82.85% (*n* = 58) completed it twice. The ICC for the overall K-DMSES score was 0.85 (*P* < 0.001; 95% confidence interval = 0.75–0.91), reflecting a satisfactory test-retest reliability. ICCs for the nutrition, physical exercise/body weight, medical treatment, and blood sugar subscales were 0.87, 0.78, 0.62, and 0.88, respectively.

## 4. Discussion

This study translated the DMSES into Korean and evaluated its psychometric properties in Korean type 2 diabetes patients. The psychometric properties of the culturally adapted K-DMSES were satisfactory. The total number of items in the K-DMSES was 16, which is fewer than in all other language versions of the DMSES except for the UK-English version, which comprises 15 items [22]. A shorter K-DMSES may represent a smaller burden for patients with type 2 diabetes, rendering it more feasible to use in practice.

Translation and back-translation of a questionnaire requires not only literal translation but also social/cultural adaptation. In this study, item 18 of the K-DMSES was changed to “… four times a year to monitor my diabetes,” based on the guidelines of the Korean Diabetes Association. A similar change was also made in the Taiwanese/Chinese version [25], in accordance with Taiwanese regulations of the Bureau of National Health Insurance. In the UK version, the item was deleted based on the National Health Service (such as GP care system in the UK) [22]. The inclusion or wording of item 18 may depend upon the prevailing health system or health policy in the country in which the questionnaire will be used.

Item redundancy on the DMSES is constantly being discussed. McDowell et al. [23] reported strongly correlated items (items 2/3, 8/11, 13/14, 13/15, and 14/15) in the Australian-English version. Sturt et al. [22] also noted duplicated items (items 4/5, 5/10, 13/14, and 13/15) in the content validity of the UK-English version. Similarly, redundancy of items 4/5, 13/14, and 15/16 was found in the K-DMSES for the content validity and the zero-order correlation matrix of items. If items of a scale are strongly correlated, it is recommended that the redundant ones should be dropped. This prevents a methodological problem with multicollinearity [38].

Factorial construct validity in this study demonstrated that the K-DMSES comprises four subscales. The items clustered into each subscale were similar to those of the Taiwanese/Chinese version [25]. The Dutch version also comprises four subscales, wherein the clustered items on the physical exercise and blood sugar subscales were similar to those of the two aforementioned versions, but the items on the other two subscales (“nutrition specific and weight” and “nutrition general and medical treatment”) were clustered differently [21]. This finding in the study of the Dutch version may be attributable to the use of an insufficient sample size (*N* = 94) for a principal component analysis. An inadequate sample size was also a weakness in the psychometric study of the Turkish version of the DMSES (*N* = 101), which revealed three subscales [24]. In addition, a single subscale was reported for the UK-English version [22], which accounted for only 41% of total variance of all items. This unidimensionality is not congruent with the assertion that diabetes management of self-efficacy is multifaceted [41]. Moreover, the total amount of variance accounted for by that unidimensionality did not meet the criterion of >50% [28].

Item 7 (“I am able to examine my feet for cuts”) has been inconsistent in its loading on factor analyses: it loaded onto the general nutrition and medical treatment subscale of the Dutch version [21], the diet/feet control subscale of the Turkish version [24], and the blood sugar/feet check subscale of the Taiwanese/Chinese version [25]. Furthermore, the item was statistically deleted in the present study. This lack of consistency may be due to there being only one item related to the confidence of foot care in the DMSES, with this item possibly being treated as relatively heterogeneous, resulting in it being statistically clustered onto various subscales, or even deleted from the scale. If there were more items related to item 7, its own subscale might have been constructed. Given that at least three items are required for a latent construct [42], it is recommended that two items should be added in future studies, for example, “confident of protecting my feet from hot and cold” and “confident of putting on shoes and socks at all times.”

Only EFA has been performed to evaluate the factorial construct validity of the DMSES—CFA has never been performed. This is the first study in which both EFA and CFA have been performed to validate the DMSES, applying a cross-validation approach. This approach has the merit of exploring the underlying construct of the items and simultaneously confirming the stability of those underlying constructs [43]. In the present study the four-subscale construct extracted from the EFA was empirically supported by CFA. However, the CFA revealed that there was covariance between the error terms of three pairs of items, items 9/10, 14/16, and 16/17, implying the presence of an unknown systematic error. Byrne [39] reported that a systematic error may occur due to an overlap in the content of items. The contents of the three pairs of items all related to “eating-related confidence.” Therefore, further study is needed to remove the possibility of content overlap.

Construct validation by means of the hypothesis testing approach refers to the correlation with one or more well-established instruments, based on a prior hypothesis [44]. The present study has demonstrated the construct validity of the K-DMSES, with a moderate correlation with the SDSCA. The Taiwanese/Chinese version of the DMSES exhibited a similar correlation (*r* = 0.58) to the SDSCA [25].

A Cronbach's alpha value of between 0.70 and 0.95 indicates sufficient item correlations and a low redundancy of items [28]. Overall Cronbach's alpha was a little higher for the K-DMSES (0.92) than for the Dutch version (0.81) [21], the UK-English version (0.89) [22], and the Turkish version (0.88) [24] and was similar to that of the Australian-English version (0.91) [22] and the Chinese version (0.93) [25]. Together these findings suggest that the DMSES has a good internal consistency across languages.

Test-retest reliability refers to the temporal stability of a scale between two time points, and the most commonly used criteria for evaluating this parameter are Pearson's *r* or ICC > 0.70 [40]. Pearson's *r* for the test-retest reliability ranged from 0.76 to 0.86 for the Dutch [21], Australian-English [22], and Chinese [25] versions. However, Pearson's *r* is criticized for being insufficiently rigorous for assessing reliability. It does not consider systematic differences as a part of measurement error, so Pearson's *r* value is usually higher than the ICC. The ICC is considered a more reliable parameter for continuous variables [28] and so was calculated in the present study, yielding a value of 0.85, which is higher than that of the UK-English version (0.77) [22] and lower than that of the Turkish version (0.91) [24]. These findings suggest that the overall test-retest reliability of the DMSES is stable over time across languages. However, the medical treatment subscale in this study was characterized by a relatively low ICC (0.62). Similarly, the temporal stability of that subscale was unsatisfactory in the Taiwanese/Chinese version (*r* = 0.69) [25]. Other studies have determined only overall values, not values for the subscales, so it is currently difficult to determine why the medical treatment subscale lacks stability.

A limitation of this study is the lack of a responsiveness test to detect changes when patients improve or deteriorate [45]. A longitudinal study should therefore be conducted which assesses the K-DMSES scores of patients in whom changes are expected to occur.

Regarding test-retest reliability, the time interval between repeated measures should be justified. In general, it is preferable for the time interval to be sufficiently long to prevent recall, but short enough so as to ensure that a clinical change has not occurred [28]. Diverse time intervals have been applied in reliability testing of the DMSES: 10 days (present study), 2 weeks [25], 3 weeks [23], 4 weeks [22, 24], and 5 weeks [21]. One empirical study found no significant differences in the test-retest reliability of health-status instruments when time intervals of 2 days and 2 weeks were applied [46]. More studies of the optimal time interval for the test-retest reliability of the DMSES are required.

## 5. Conclusion

The K-DMSES was subjected to culture-sensitive translation and its psychometric properties were validated in Korean type 2 diabetes patients. The underlying construct of the K-DMSES comprises four subscales: nutrition (items 4, 9, 10, 14, 16, and 17), physical exercise/body weight (items 6, 8, 11, and 12), medical treatment (items 18, 19, and 20), and blood sugar (items 1, 2, and 3). The K-DMSES demonstrated good content validity, factorial construct validity, hypothesis testing construct validity, internal consistency reliability, and test-retest reliability. This instrument is ready for use in both research and practice.

## Figures and Tables

**Table 1:** General characteristics.

Variable	Subsample 1 (*n* = 220) *n* (%)	Subsample 2 (*n* = 220) *n* (%)	*χ* ^2^ or Fisher's exact test (*P*)
Gender			0.146 (0.703)
Male	111 (50.5)	115 (52.3)	
Female	109 (49.5)	105 (47.7)	
Age (years) (mean ± SD = 58.02 ± 0.88)			3.902 (0.561)
20–29	2 (0.9)	3 (1.4)	
30–39	7 (3.2)	10 (4.5)	
40–49	34 (15.5)	30 (13.6)	
50–59	87 (39.5)	71 (32.3)	
60–69	57 (25.9)	68 (30.9)	
≥70	33 (15.0)	38 (17.3)	
Marital status			0.705 (0.894)
Married/living together	178 (80.9)	173 (78.6)	
Divorced/widow(er)	32 (14.5)	37 (16.8)	
Unmarried	9 (4.1)	9 (4.1)	
Other	1 (0.5)	1 (0.5)	
Job			2.142 (0.295)
Employed	102 (46.4)	114 (51.8)	
None	117 (53.1)	106 (48.2)	
Data missing	1 (0.5)	0 (0.0)	
Education			1.230 (0.873)
Elementary school	34 (15.5)	35 (15.9)	
Middle school	30 (13.6)	32 (14.5)	
High school	88 (40.0)	88 (40.0)	
College and above	59 (26.8)	60 (27.3)	
Other	9 (4.1)	5 (2.3)	
Monthly income (KRW)			1.147 (0.766)
Less than 2,000,000	88 (40.0)	79 (35.9)	
2,000,000–2,999,999	40 (18.2)	39 (17.7)	
3,000,000–3,999,999	33 (15.0)	40 (18.2)	
4,000,000 and above	56 (25.4)	54 (24.5)	
Data missing	3 (1.4)	8 (3.6)	
Treatment regimen			1.164 (0.762)
Diet/exercise only	9 (4.1)	7 (3.2)	
Oral hypoglycemic agent	141 (64.1)	151 (68.6)	
Insulin	10 (4.5)	10 (4.5)	
Oral hypoglycemic agent + insulin	60 (27.3)	52 (23.6)	
HbA1c (mean ± SD = 7.70 ± 1.38)			0.011 (0.918)
Controlled (HbA1c < 7.0%)	69 (31.4)	70 (31.8)	
Uncontrolled (HbA1c ≥ 7.0%)	151 (68.6)	150 (68.2)	

HbA1c: hemoglobin A1c; KRW: South Korean won.

**Table 2:** Exploratory factor analyses.

Number	Abbreviated item description	First exploratory factor analysis^a^				Second exploratory factor analysis^b^			
		F1^c^	F2^d^	F3^e^	F4^f^	F1^c^	F2^d^	F3^e^	F4^f^
1	Checking blood sugar				0.46				0.46
2	Correcting high blood sugar				0.80				0.84
3	Correcting low blood sugar				0.83				0.81
4	Choosing foods	0.52				0.53			
6	Controlling body weight		0.59				0.58		
7	Examining feet for cuts					—	—	—	—
8	Taking physical exercise		0.74				0.74		
9	Adjusting eating plan during illness	0.53				0.54			
10	Following a healthy eating pattern	0.67				0.57			
11	Taking physical exercise on doctor's advice		0.84				0.81		
12	Balancing between exercise and eating plan		0.74				0.74		
14	Adjusting eating plan: when I am away from home	0.75				0.76			
16	Eating pattern: eating out, eating at a party or company dinner	0.80				0.79			
17	Eating plan related to stress or anxiety	0.76				0.74			
18	Visiting doctor four times a year			0.73				0.70	
19	Taking medication as prescribed			0.87				0.91	
20	Adjusting medication during illness			0.74				0.73	

^a^First exploratory factor analysis: Kaiser-Meyer-Olkin (KMO) statistic = 0.89, Bartlett's sphericity *χ* ^2^ = 2602.62 (*P* < 0.001).

^b^Second exploratory factor analysis: KMO statistic = 0.89, Bartlett's sphericity *χ* ^2^ = 2461.10 (*P* < 0.001).

^c^Factor 1: nutrition.

^d^Factor 2: physical exercise/body weight.

^e^Factor 3: medical treatment.

^f^Factor 4: blood sugar.

**Table 3:** Summary of fit indices from confirmatory factor analysis.

	*χ* ^2^	df	CMIN/DF	GFI	SRMR	RMSEA (90% CI)	CFI	NFI
Model 1	391.57^*^	98	3.99	0.81	0.07	0.12 (0.10–0.13)	0.87	0.84
Model 2	325.06^*^	97	3.35	0.85	0.06	0.10 (0.09–0.11)	0.90	0.97
Model 3	284.50^*^	96	2.96	0.87	0.06	0.09 (0.08–0.10)	0.92	0.88
Model 4	253.11^*^	95	2.66	0.88	0.06	0.08 (0.07–0.10)	0.93	0.90

df: degrees of freedom; CMIN/DF: ratio of *χ* ^2^ value to the degrees of freedom; GFI: goodness-of-fit index; SRMR: standardized root-mean-square residual; RMSEA (90% CI): root-mean-square error of approximation with 90% of confidence interval; CFI: comparative fit index; NFI: normed fit index.

^*^ *P *< 0.001.
